# The Connection between Neurophysiological Correlates of Trust and Distrust and Isolated HEXACO Dimensions

**DOI:** 10.3390/life14030362

**Published:** 2024-03-09

**Authors:** Dimitrios Külzer, Stefan Kalt, Peter Walla

**Affiliations:** 1 Faculty of Psychology, Freud CanBeLab, Sigmund Freud University Vienna, Freudplatz 1, 1020 Vienna, Austria; dimitrios.kuelzer@sfu.ac.at (D.K.); stefan.kalt11@gmail.com (S.K.); 2 Faculty of Medicine, Sigmund Freud University Vienna, Freudplatz 3, 1020 Vienna, Austria; 3 School of Psychology, Newcastle University, University Drive, Callaghan, NSW 2308, Australia

**Keywords:** electroencephalography (EEG), event-related potential (ERP), trust, distrust, HEXACO, agreeableness, honesty–humility, neuroconsulting, marketing

## Abstract

Trust and distrust are constructs that have provoked and undergone lots of discussion in the fields of sociology and psychology. However, to our knowledge, there is little agreement about how these constructs should be treated in the future. The present study tries to help in this discussion by re-analyzing prior neurophysiological data highlighting differences between trust and distrust by connecting these data with two distinct personality dimensions. Thus, the objective was to analyze the connection between neurophysiological trust/distrust processing and distinct HEXACO personality dimensions. Differences were found in the event-related potentials (ERPs) calculated for visual presentations of political institution words and brand names, which were evaluated with respect to trust and distrust by button presses. Two time points (330 ms and 780 ms) showed brain activity differences between trust and distrust related to the two word categories at frontal electrode locations. For this study, these findings were taken and connected to HEXACO-60 personality inventory results collected from prior participants. Statistical analysis revealed a significant interaction between the ERPs and two HEXACO personality dimensions concerning trusted brands at the later time point (780 ms) at the right frontal electrode location F8. This result is taken as neurophysiological evidence that parameter values of the personality traits honesty–humility and agreeableness have an influence on brain functions related to trusted brands.

## 1. Introduction

Since the 1950s, the constructs of trust and distrust have been discussed in the fields of psychology and sociology [1,2,3]. Initial analyses, like the differentiated theory of [2] or the psychoanalytic hypotheses by Erik H. Erikson (1953, cited by [1]), usually only highlight the construct of “trust” (due to their belief that “distrust” only states the opposite of trust) as a situational and dynamic psychological decision-making process. Newer theories depict trust and distrust as two independent constructs, which interact with each other and are not only created from situationally based evaluations, but also from dispositive tendencies [4]. Furthermore, specific experiences (i.e., interindividual contact) seem to impact trust and distrust, an idea which has developed over time [5,6]. The initial concept was often revised, and according to multiple different paradigms and approaches, trust and distrust seem to be highly heterogenic in their theoretical conception, which impedes a holistic taxonomy as well as a possible objective operationalization. Especially in modern psychological research, “trust” and “distrust” are embraced as transactional cognitive information-processing functions, which are utilized to reduce the complexity of exogenetic factors and augment safety [4,7]. Based on these concepts, the present paper aims to apply newer transactional theories related to the neurophysiological correlates of trust and distrust by adding two arguably relevant personality dimensions.

In modern neuroscience, trust and distrust are rarely investigated. Most of the little existing literature is focused on localizing neural correlates for these constructs [8,9,10,11,12] or concentrated on finding specific genes, neurotransmitters, and specific hormone levels interfering with the endocrinal system, which correlate with trust [13,14]. One of these studies was conducted by [9], who reported to have found different anatomical correlates for trust- and distrust-related processes by introducing an economic decision-making test. Ref. [9] defines anatomical regions, like the nucleus caudatus and the putamen (both parts of the basal ganglia), as well as parts of the dopaminergic rewarding system as localizable regions for trust-related processing. In contrast to these findings, the limbic system, especially the amygdala, but also the insular cortex, could be associated with distrust. With these analyses, ref. [9] could initially show, on a neurobiological level, that trust and distrust seem to be separate constructs, which was already theoretically assumed [13,15]. Besides studies concentrating on optical brain imaging, some studies have elaborated social interactions with electrophysiological methods using, at least partly, constructs like trust and distrust [16,17,18]. For instance, ref. [18] measured the influence of trust violations on working memory and to what extent emotions interfere with this process. The respective results suggested that trust violations reduce response velocities and inhibit the actualization processes of emotional information. Even though [18] partly elaborated on the construct of trust, there are, to the best of our knowledge, no distinctive studies that have analyzed the processing of trust and distrust through electroencephalography (EEG) methodology, other than a very recent prior study that formed the basis for the present paper [19]. In that study, it was found that brain activity differences between trust and distrust even depended on the word stimulus category. Visual presentations of brand names and names of political institutions had to be responded to with respect to individual trust or distrust. At an early time point of around 330 ms post-stimulus onset, event-related potentials (ERPs) in the left frontal region varied as a function of trusted or distrusted political institution stimuli, whereas the ERPs in the right frontal region at a later time point of around 780 ms post-stimulus onset varied as a function of trusted or distrusted brand names. This detailed hemispheric lateralization finding has been interpreted as neurophysiological evidence for the complex nature of the trust and distrust constructs. These promising ERP findings were taken as the basis for the present study, following the hypothesis that distinct personalities have an influence on brain activity related to trust and distrust.

Personality research suggests that specific dispositive dimensions correlate with higher or lower trust tendencies [20,21,22]. For example, ref. [21] introduced the facet of trust as a sub-facet of agreeableness in their NEO-PI-R questionnaire. Ref. [22] defined trust as a stable and consistent personality trait, which should be considered separately. This and other theories even inspired scholars trying to develop newer trust and distrust theories by incorporating dispositive tendencies in their framework, like the differential trust and distrust theory by [4] or the trust model by [23]. The assumption that personality and trust/distrust at least interact with each other is not new. Nevertheless, little to no research has elaborated on personality traits and the constructs of trust and distrust by utilizing neurophysiological technology. Due to these circumstances, the current exploratory study aims to first establish findings in this interconnected field of social psychology, neuroscience, and personality science.

## 2. Research Question and Hypotheses

As shown in the introduction, the neurophysiological elaboration of trust and distrust as well as the connection of these constructs with established personality factors has seldomly been analyzed and seems to be a unique, but complex proceeding that needs specific clarification. Furthermore, findings of the replication crisis in psychology accentuate the high priority of transparent scientific work [24], which include a precise composition of the methodical procedure, as well as a differentiated representation of the elaborated steps. A short, but detailed presentation of the research question and hypotheses follows.

The aim of the present study is a neurophysiological exploration of the previously only psychometrical (i.e., [4,25,26]) and neuroanatomical (i.e., [9,10]) distinctions between trust and distrust. We expected to find significant differences in neurophysiological trust/distrust processing depending on the expression level of the HEXACO personality dimensions of honesty–humility and agreeableness. This is carried out by generating and analyzing event-related potentials (ERPs) and correlating them with specific personality profiles. The authors of this paper used the HEXACO model by [27] for the analysis, which represents an updated version of the OCEAN model by [21], which introduced the new sixth personality factor, honesty–humility. Due to the theoretical conception of the personality dimensions of agreeableness and honesty–humility [20,21] and the fact that authors like [4,22,23,28] highlighted the dispositive aspects of the constructs of trust and distrust, the authors of this paper decided to focus on the facets of agreeableness and honesty–humility for their initial explorative analysis. Furthermore, ref. [29] assumes that younger people show higher dispositive trust tendencies than elderly people due to their cognitive development level. This hypothesis was formulated after the author showed, in a developmental psychology study, that younger persons with high trust tendencies perceive exogenetic factors as significantly more trustful than other populations do. Due to these results, the authors of this study opted to highlight the developmental stage of emerging adulthood (18–24 years), which was defined by [30], as well as young adulthood (24–34 years) by [31] to include [29]’s assumption. 

The main hypotheses can be defined as follows:

**H0a:**  *There is no significant differentiation between the constructs of trust and distrust concerning specific neuronal potentials at exact timeframes for emerging and young adults.*

**H1a:**  *There is significant differentiation between the constructs of trust and distrust concerning specific neuronal potentials at exact timeframes for emerging and young adults.*

**H0b:**  *There is no significant differentiation between the personality factors of agreeableness and honesty–humility concerning the neuronal processing of the constructs of trust and distrust in specific neuronal areas at exact timeframes for emerging and young adults.*

**H1b:**  *There is significant differentiation between the personality factors of agreeableness and honesty–humility concerning the neuronal processing of the constructs of trust and distrust in specific neuronal areas at exact timeframes for emerging and young adults.*

To answer these hypotheses adequately, it was important to formulate sub-hypotheses and analyze different subsystems that contribute to the researched phenomena. Besides these, the authors assessed descriptive statistical data, like age, sex, educational level, and specific job qualifications, which are stated in the following chapters as well.

## 3. Materials and Methods

Before the results of the formulated research question and hypotheses are expounded, it is of utmost necessity to elucidate the different operationalizations and the experimental procedure. Furthermore, the experimental design and the sampling method are presented in detail. Additionally, the different behavioral and physiological measurements are explained, as well as how their data were assessed.

### 3.1. Sampling

Initially, 40 participants were invited to the Freud CanBeLab (Freud Cognitive & Affective Neuroscience and Behaviour Lab), where sociodemographic, behavioral, and neurophysiological data were collected. This test group was recruited ad hoc by snowball [32] and convenience-manner sampling [33]. Due to uncontrollable measurement artifacts, 3 of the 40 participants had to be excluded. The remaining 37 participants, consisting of 19 males and 18 females with ages ranging from 18 to 34 years (M = 22.92, SD = 3.26), were introduced to the experiment via a written information statement. None of the participants reported a neuropathological or psychopathological history. The educational levels of the participants were as follows: 28 people (75.7%) had A-levels, 8 people (21.6%) had a higher education degree (university, college, university of applied science, etc.), and only 1 person (2.1%) had only completed compulsory education. Regarding the replication crisis in psychology, we wanted to counteract the statement that psychological experiments only consist of psychology students. For the current experiment, only 9 participants (24.3%) were studying or studied psychology. Furthermore, zero people stated frequent or current drug abuse, which was an exclusion criterion due to inducing diminished vigilance and longer reaction latencies. Every participant professed no somnolence and were well rested. All participants were right-handed and had normal or corrected to normal vision. They signed a consent form and were informed that they could withdraw from the study at any time during the experiment without any consequences (see [19]).

### 3.2. Stimuli

The used word lists consisted of 80 political institutions (only Austrian institutions) and 80 randomly picked brand names (commonly known brands) (see Appendix A, Table A7). The decision of which specific stimuli should be used for this experiment was based on a small pre-test sample of n = 5, in which the words of a list of over 600 institutions seemed to be relevant in their minds, resulting in a list of about 250 items. Of these, we pseudo-randomly selected 120 institutions. Every stimulus was shown for 0.3 s with a 1 s pause in between. Every pause presented a fixation cross in the middle of the screen with the purpose of guiding the gaze of the participants to the visual area of interest. These stimuli were presented using the open-source software PsychoPy 3 for Windows 11 (Version: 3 February 2021) [34]. The stimuli were shown in a randomized order, and each stimulus was presented twice to increase the number of usable stimuli for the generation of ERPs later in the process. The programmed software scripts in PsychoPy were designed to also send triggers to the EEG recording software to provide condition coding for later EEG and ERP data analysis. For each stimulus presentation, the participants had to respond via a button press whether they felt trust or distrust related to the specific institution or brand or whether they were not sure or did not even know the institution or brand (see [19]).

### 3.3. Electroencephalography (EEG)

For the neurophysiological measurements, we used the gel-based 64-channel multipolar actiCHamp Plus System from Brain Products with active electrodes embedded in an actiCAP connected to an amplifier. The amplifier was operated by a lithium-ion battery pack. The electrodes were applied according to the international 10–20 system [35,36]. Brain potentials were sampled at a rate of 1000 Hz (filtered: DC to 100 Hz). Impedance was kept equal to or below 5 kΩ. The Cz electrode was used as the reference electrode, and a mid-frontal position on the forehead was used as the ground electrode. The collected data were then processed offline using EEGDISPLAY (Version: 6.4.9), which was developed by [37]. First, all data were down-sampled to 250 Hz. Then, a bandpass filter ranging from 0.1 Hz to 30 Hz was applied. Epochs were generated from −100 ms before stimulus onset (baseline) to 1000 ms after stimulus onset. All data were baseline-corrected (−100 ms to 0 ms). All epochs with visible artifacts were excluded from further analysis. ERPs were generated from the cleaned data for all 4 conditions (trusted and distrusted political institutions and trusted and distrusted brands), every electrode, and each participant. For further statistical analysis, ERPs with amplitudes of ±75 µV were excluded. For the purpose of statistical analysis, the data were further down-sampled (averages across 40 ms) and exported to the statistics software IBM SPSS Statistics 27 (Version 27.0; 64-Bit), which was used for interference statistical analysis of the data. To display the ERP findings in connection with personality scores, grand-average ERPs were generated across all members within a distinct personality score level.

### 3.4. HEXACO-60

For the examination of the personality profile, we used the German version of the HEXACO-60 by [38], which was validated and reliability-tested by [39]. The HEXACO-60 consists of 60 items, with each of the six facets (honesty–humility, emotionality, extraversion, agreeableness, conscientiousness, and openness to experience) each containing 10 items. The respective Cronbach’s alpha values of the HEXACO-60 dimensions are α = 0.84 for the honesty–humility dimension, and α = 0.76 for the agreeableness dimension [40]. The questionnaire provides a Likert-scale with a single-choice five-options-response system (from strongly disagree to strongly agree). The assessment of the test was conducted by summarizing the raw item data and dividing these by 10, which represents the value of each facet. Finally, these findings were compared with the descriptive norm-statistics by [40] for German-speaking countries, which allowed for categorization of the data into low, normative, and high scores. The evaluation of the test results was calculated manually and transferred to IBM SPSS Statistics (Version 27.0; 64-Bit).

### 3.5. Experimental Design

**Figure 1:**
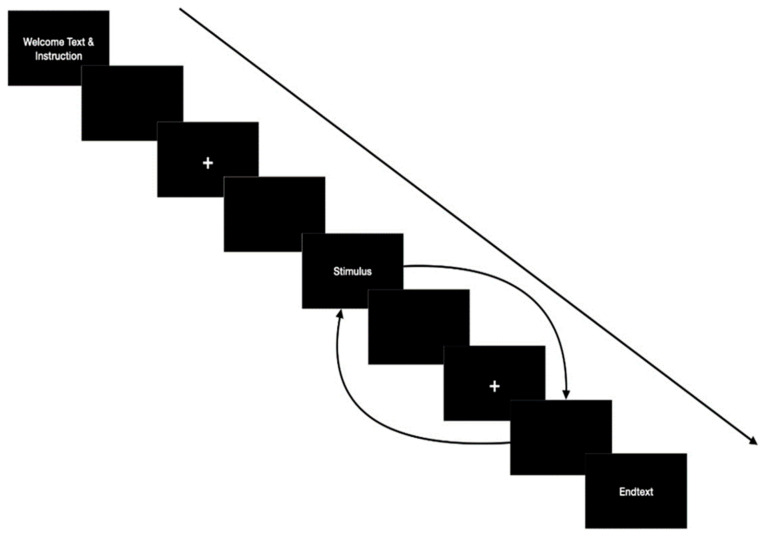
Sequence of events presented during the experiment. A fixation cross presented for 1 s was followed by a blank screen shown for 0.5 s, and then a word stimulus was presented for 0.3 s, followed by a further blank screen for 1 s. See [19].

Each participant that arrived at the Freud CanBeLab was initially given a form with general information about the study, a consent form, a sociodemographic survey, and the HEXACO-60 Personality Inventory. These could be completed without any time restrictions, and potentially misleading details were clarified. After the documents were collected, the installation of the EEG according to the above-mentioned criteria (see Section 3.3) was conducted. While applying the gel, impedance was observed via BrainVision Recorder software (Version: 1.23.0003). The experiment itself was designed as an RSVP (rapid serial visual response) paradigm [41], in which a single word was shortly shown, followed by a response cue from the participant. Then, a fixation cross was shown before the next word appeared. When reaching the desired impedance, a verbal instruction was given to the participants, advising them to sit still during the presentation of the stimuli and to only blink when the fixation cross was shown (see Figure 1). The time interval for one trial was 1000 ms for the fixation cross, 500 ms for the blank screen and 300 ms for the respective stimulus presentation (see [19]). After one trial, that is, for each shown stimuli, the participants were asked to respond according to their subjective feeling of trust or distrust toward each stimulus by pressing one of the arrow keys on a standard keyboard. In cases where the participant could not reach a conclusion or was not familiar with a shown stimulus, another arrow key was pressed. These data were excluded from further analysis. The experiment was carried out using a 28-inch monitor for the visual presentations with a viewing distance of 1 m. The EEG activity during the experiment was observed by only one person. The total time of the experiment, including the EEG setup, was between 1.0 and 1.5 h.

### 3.6. Statistical Analysis

For answering the formulated hypotheses, we initially re-analyzed the findings of [19] by conducting matched-pair *t*-tests and Wilcoxon tests on the constructs of trust and distrust. These allowed us to reassess their findings and answer the first main hypothesis. The second main hypothesis was evaluated by computing an overall MANOVA (multivariate analysis of variances) with respective ANOVAs. Finally, we calculated Benjamini–Hochberg corrections [42] to prevent alpha inflation [43]. For all inference statistical methods, the required conditions were calculated as well, meaning that the covariance matrices of the MANOVA, the variance homogeneity of the deducted ANOVAs, the normal distribution, and the corresponding graphical interactions of the ANOVAs were assessed. Additionally, we evaluated the normal distribution of the matched-pair t-tests, which showed us the necessity of using a Wilcoxon test on some of the data [32]. All calculated statistical outputs can either be found in the text or in the Appendix A.

## 4. Results

The following section presents the different data and statistical analyses that were calculated. For this, we initially present the results of the averaged personality profiles of the participants. Thereafter, statistical analysis of the neurophysiological data follows, and a conclusion is given by formulating an answer for the defined hypotheses.

### 4.1. Results of the HEXACO-60

The HEXACO-60 profiles showed diverse and highly individual values of the different personality variables, which revealed complex interactivities between the factors. However, due to the focus of this study on the personality traits of honesty–humility and agreeableness (see introduction) as well as on the economic criterion of quality, only these two facets are examined here.

#### 4.1.1. Honesty–Humility

Concerning the facet of honesty–humility, according to [40], the mean for German populations is 3.52 (SD = 0.58). A total of 29 people (78.4%) (18 male and 11 female) showed a normative value. Only one female participant (2.7%) could be categorized in the low-value category, while seven persons (18.9%) (one male and six females) showed a facet score higher than the average population. Concerning their educational level, 23 individuals (62.2%) of the normative-scoring group had completed their A-levels, 1 person (0.3%) had completed compulsory school, and 5 participants (0.14%) attended higher-education institutions. The one female participant who showed lower honesty–humility scores than the standardized group by Ashton et al. (2006) attended a higher-education institution. Out of the seven individuals who scored higher than normative, five persons (0.14%) had accomplished their A-levels, and two persons (0.05%) attended higher-education institutions. Furthermore, only 1 (2.7%) of the 9 participants who were studying or studied psychology showed higher value scores in honesty–humility than the average population, while the other 8 persons (21.6%) could be found in the normative group. In contrast, out of the 28 persons who were not or did not study psychology, only 1 person (2.7%) showed lower-value scores than average, and 6 participants (16.2%) had higher results. A total of 21 individuals (56.7%) of these 28 persons were categorizable as normative in honesty–humility. Overall, in this study, the mean score of the facet of honesty–humility was 2.16 (SD = 0.44). In Table 1, the results are visualized.

#### 4.1.2. Agreeableness

Concerning the facet of agreeableness, according to Ashton et al. (2006), the mean for German populations is 2.99 (SD = 0.45); 23 subjects (62.2%) reached a normative value. The rest of the sample seemed to be evenly distributed over the low (seven persons, 18.9%) and high value category (seven persons, 18.9%). Furthermore, 4 male and 3 female participants could be categorized into the low agreeableness scored group, while 12 male and 11 female participants scored in the normative range. Only four male and three female subjects were categorizable into the high-scoring agreeableness group. Focusing on their educational level, 23 participants (62.1%) who completed their A-levels showed a normative agreeableness profile. In contrast, only five persons (13.5%) with an A-level degree scored higher than the norm, while none scored lower. Additionally, five persons (13.5%) who attended higher-education institutions showed normative agreeableness scores, one participant (0.3%) with a higher educational degree maintained a low agreeableness score, and two subjects (0.54%) of the same educational level seemed to possess higher scores than the normative interval. The one person who only completed compulsory school showed normative values of agreeableness. Finally, the agreeableness profile of this sample illustrates that 20 persons (54.1%) of the 28 non-psychologists showed normally distributed scores for this facet. Out of the remaining eight participants (21.6%) of this subgroup, four subjects were categorizable in the lower-scoring group, while the other four participants could be found in the higher-scoring group. The opposing group of the nine persons (24.3%) who were or did study psychology were evenly distributed over the three possible outcomes (low, normal, or high agreeableness values). Finally, the mean score of this sample for the facet agreeableness was 2 (SD = 0.62). See Table 2 for all results.

### 4.2. Inference Statistical Results

For answering the initial hypothesis (Ha), we reanalyzed the findings by Walla et al. (2023) by conducting matched-pair t-tests of trust × distrust with the visually inspected electrodes (F7, F8, P7, P8) at specific timestamps (330 ms, 780 ms) (see Table 3). After that, the second hypothesis (Hb) was statistically analyzed by using a multivariate general linear model (mGLM), also known as MANOVA (multivariate analysis of variances) [32] by testing if the personality variables of honesty–humility and agreeableness (independent variables; IV) differed electrophysiologically between trust and distrust (dependent variables, DV). Due to the fact that we explored multiple sub-hypotheses to answer our two main-hypotheses, we had to correct for alpha inflation. We decided to introduce a Benjamini–Hochberg correction (Pi ≤ i/m Q) [42] due to it being more progressive than the classical conservative alternative Bonferroni correction but still essentially reducing the false-positive rate (FDR). Hence, a false-positive rate (Q) of 5% was defined for each ranked hypothesis (i) out of a total of 31 calculations (m) (2 main-hypotheses and 29 sub-hypotheses). The BH correction is provided in the Appendix A.

Beginning with the analysis of the first formulated main hypothesis, it is highly important to test the statistical requirements before conducting a t-test. As stated by [32,44] a matched-pair *t*-test requires an assessment of the normal distribution of the sample for optimal validity. Thus, the normal distribution was calculated using the KS (Kolmogorov–Smirnov) and SW (Shapiro–Wilk) verifying tests. For this research question, the neurophysiological data of trust and distrust had to be calculated as matched pairs, so difference scores were created before conducting the test for normal distribution as a standard procedure [32,44]. This indicates that the ERPs for trust and distrust on each selected electrode at the important time points were subtracted from each other, resulting in the necessary data for the requirement check (see Appendix A). From this analysis, it is observable that not all difference scores fulfilled the requirement of normal distribution. Hence, good statistical practice recommends analyzing these electrophysiological data with an alternative, non-parametric test [32,44]. For this, the Wilcoxon test was chosen (see Table 4), while the potentials that did not violate the requirements were analyzed by matched-pair *t*-tests.

The results of the matched-pair *t*-test show significant differences between politically induced trust and distrust at the electrode position F7 (frontal 7) at 330 ms (t(36) = 3.03, *p* = 0.005, d = 0.5). Therefore, H0a can be discarded. After correcting with Benjamini–Hochberg, the *p*-value stayed significant, due to being smaller than the critical BH value (*p* = 0.005 ≤ 0.014). The remaining *t*-tests showed no significant distinctions between the constructs of trust and distrust at the visually inspected electrode positions (*p* > 0.05). Furthermore, the analysis of the Wilcoxon test data depicts the following.

The results of the non-parametric Wilcoxon test suggest a significant difference between the constructs of trust and distrust evoked by economical words at 780 ms on the frontal lobe (frontal 8) of the right hemisphere (Z = −2.63, *p* = 0.008). Additionally, the Wilcoxon test showed a significant difference between politically induced trust and distrust at the electrode position P7 (parietal 7) at 330 ms (Z = −2.11, *p* = 0.035). Therefore, H0a can be discarded. When applying Benjamini–Hochberg correction to these two significant sub-hypotheses, only the first one could withstand the correction (*p* = 0.008 ≤ 0.014), while the second one showed higher *p*-values than the critical BH value (*p* = 0.035 > 0.014). The remaining non-parametric tests showed no significant findings (*p* > 0.05).

Finally, inference statistical analysis of the second main hypothesis (Hb) was conducted. For this, a MANOVA (multivariate analysis of variances) was calculated, in which the interindividual honesty–humility and agreeableness personality profiles of this sample were tested concerning a distinction between the neurophysiological potentials of trust and distrust (note: for this, we used economic and political trust and distrust potentials from the electrodes F7 and F8 at the timestamps of 330 ms and 780 ms) (see Table 5). As stated by Zöfel (2003), the statistical requirements before computing the MANOVA must be checked. For this, the multivariate normal distribution and the homogeneity of the covariance matrices must be given. The assessment of the requirements showed that the homogeneity of the covariance matrices was not violated, in contrast to the multivariate normal distribution (see Appendix A). This means that the MANOVA showed a lower sensitivity. Lastly, between-subject ANOVAs were initiated. All calculations were corrected by the Benjamini–Hochberg method [42].

After analyzing the MANOVA, it is observable that honesty–humility (Λ = 0.28, F(8,54) = 5.99, *p* < 0.001), agreeableness (Λ = 0.18, F(8,54) = 9.22, *p* < 0.001), and the interaction of the two personality variables showed significant differences between the electrophysiological constructs of trust and distrust at the frontal lobe (electrodes F7 and F8) at 330 ms and 780 ms (Λ = 0.16, F(8,54) = 10.19, *p* < 0.001). Therefore, H0b can be discarded. These results could withstand Benjamini–Hochberg correction (*p* < 0.001 ≤ 0.014).

Subsequently, between-subject ANOVAs were carried out to further explore the significant findings of the MANOVA (see Table 6). For this, the requirements of an ANOVA (normal distribution and variance homogeneity) were examined, which can be found in the Appendix A.

The findings of the between-subject ANOVAs state that, when only focusing on the personality factor of honesty–humility, significant differences among the low, normative, and high values can be found in the neurophysiological processing of trust, evoked by economical words, in the right frontal lobe (F8) at 780 ms (F(2,30) = 6.13, *p* = 0.006, η^2^ = 0.29) and economically induced distrust in the right frontal lobe (F8) at 780 ms (F(2,30) = 4.81, *p* = 0.015, η^2^ = 0.24). Furthermore, by only centralizing the personality facet of agreeableness, economically induced trust processing at the right frontal lobe (electrode F8) at 780 ms seemed to differ significantly among the low, normal and high agreeableness scores (F(2,30) = 17.94, *p* < 0.001, η^2^ = 0.55). Concentrating on the interaction between the agreeableness and the honesty–humility personality profiles, neurophysiological trust processing (economically induced) differed significantly at 780 ms on the right frontal lobe (F8) (F(2,30) = 31.75, *p* < 0.001, η^2^ = 0.68). Therefore, H0b can be discarded. Additionally, all the significant hypotheses could withstand Benjamini–Hochberg correction (*p* < 0.001 ≤ 0.014), despite the economically induced distrust processing at the right frontal lobe (F8) at 780 ms, which showed a higher *p*-value than the critical BH value (*p* = 0.015 > 0.014).

### 4.3. Event-Related Potentials (ERPs) and Topographical Maps

We first show the ERP results from a prior study by [19]. Derived firstly from theoretical assumptions by [9,10,14], four electrode locations, which represent significant neuroanatomical areas for trust and distrust processing, were chosen. These electrodes were the F7 (frontal left-sided), F8 (frontal right-sided), P7 (parietal left-sided) and P8 (parietal right-sided).

**Figure 2:**
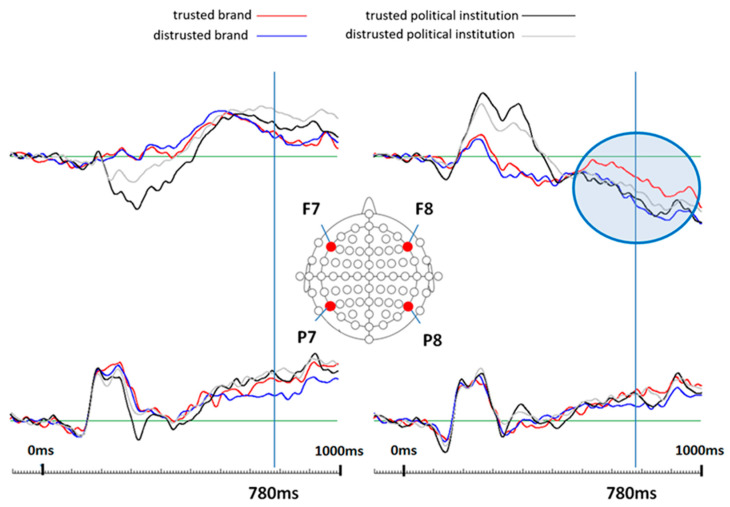
Grand-average ERPs elicited by trusted and distrusted political institutions and by trusted and distrusted brands. Note that the condition of trusted brands stands out at the right frontal electrode location F8 with the marked time point of 780 ms after stimulus onset, which showed significant differences between trusted and distrusted brands (see blue circle). This figure was adapted from [19].

Ref. [19] found that the stimulus category (i.e., political institutions vs. brand names) alone modified brain activities (ERPs) between approximately 200 ms and 500 ms after stimulus onset. Most interestingly, they also found that trust and distrust differences were different between both word stimuli categories. ERP differences related to trusted versus distrusted political institutions occurred mainly in the left frontal region between about 200 ms and 500 ms after stimulus onset (statistically significant at around 330 ms). ERP differences related to trusted versus distrusted brands occurred mainly in the right frontal region roughly between 700 ms and 900 ms after stimulus onset (statistically significant around 780 ms). These findings were reanalyzed in this study. Figure 2 shows the effects found in [19]. According to the statistical results of this study, there is a significant interaction between the personality dimensions of honesty–humility and agreeableness concerning the neurophysiological trust processing of brands at 780 ms on the right frontal lobe (electrode position F8). The blue circle in Figure 2 marks this condition for the ERP recorded at electrode location F8, which was the only location that showed a significant personality trait interaction with ERPs. As can be seen, this condition elicited the most negative ERP compared to the other three conditions across all 37 participants.

**Figure 3:**
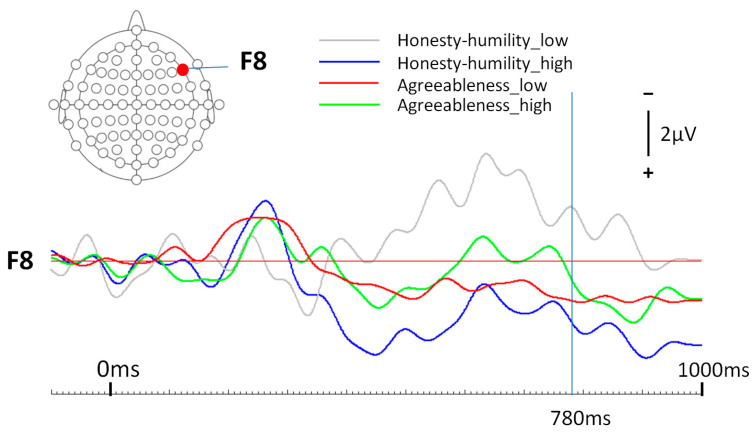
Grand-average ERPs for low and high scorers of the two personality traits of honesty–humility and agreeableness.

**Figure 4:**
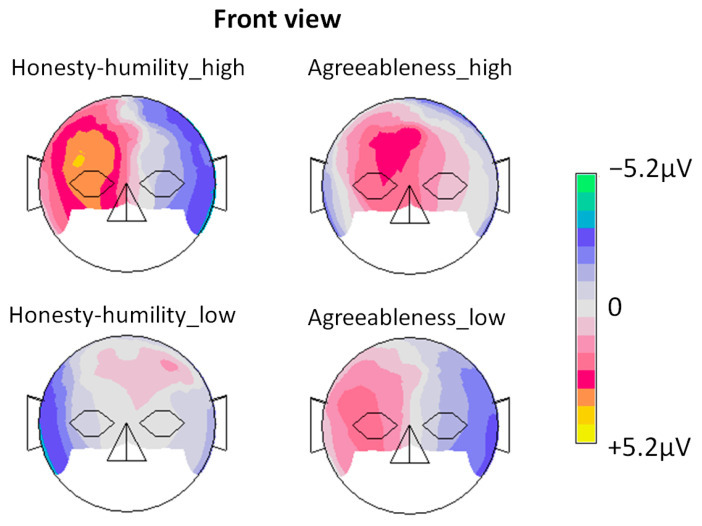
Topographical maps for all four personality groups using all electrode locations. Note that the condition of high honesty–humility elicited the most positive ERP in the right frontal region.

Finally, grand-average ERPs of this condition (trusted brands) were generated for each of the four participant groups according to the inferential statistics results shown before. Figure 3 and Figure 4 show the respective results.

## 5. Discussion

As shown by the inference statistical part of this paper, we could successfully answer the initially formulated main hypotheses and showed, as far as the researchers know, for the second time besides the initial calculations by [19], electrophysiological correlates at specific timepoints at which the constructs of trust and distrust differed. As mentioned in the introduction, the present study was meant to use these exact findings to re-analyze ERP data with respect to HEXACO personality trait results focusing on the two traits of honesty–humility and agreeableness. Furthermore, it was possible to find distinctions between the expressions of specific personality factors (honesty–humility and agreeableness) concerning the neurophysiological processing of trust and distrust. Through this, it was feasible to validate, by electroencephalographic exploration, the theoretical assumptions of modern, transactional trust and distrust theories, like those of [4] or [23], who showed psychometrically that trust and distrust are partly evoked by dispositional tendencies.

Due to the fact that this study was an explorative one, it was primarily achievable to show specific neurophysiological components that could be associated with trust and distrust processing. For instance, politically induced trust and distrust seemed to distinguish their processing at about 330 ms (EPC, early positive component), where political trust evoked higher negativity than distrust. In contrast, brand-related trust and distrust were distinct from each other later, at about 780 ms (LPC, late positive component), which implies that the distinction between trust and distrust, at least for economically institutions, could be a higher complex cognitive process, which is localized in the prefrontal lobe. These findings are consistent with the results of [9,10,13], who correlated trust processing with the dopaminergic approach system. Nevertheless, these findings also raise new questions. Why does the neurophysiological process of trust and distrust seem to differ so significantly between economically and politically evoked words? Does the political wording due to higher polarizations possess higher arousal levels than the economical notions, which further induce a more elaborated and faster processing [45]? If this consideration is supported by future research, the emotional component of trust- and distrust-related stimuli could achieve higher relevance than assumed until now. Are trust and distrust similar to the emotional face expression (EFE) paradigm [46,47,48] influenced by affective processing? Or is politically evoked trust and distrust processing more influenced by top-down processing, which would explain, at least partly, why the high negative amplitudes arise about 450 ms before brand-related processing happens? Further research is needed to explore these considerations, thus giving the paradigm of trust and distrust new explorable research fields in neuroscience.

Besides these points, the statistical analysis of the neurophysiological data as well as the visual observation showed distinctive lateralization processes between politically and economically related trust and distrust processing. Here, it was depictable that brand-related trust and distrust especially differed in electrophysiological processing on the right hemisphere, while politically induced trust and distrust potentials seemed to differ on the left hemisphere. This allows for implications and applications of the constructs of trust and distrust on modern lateralization models, like the analytic–synthetic model [49,50]. This model understands the left hemisphere as the processing-center for abilities and tasks, which need microscopic and highly differentiated toolsets, while the right hemisphere is mostly correlated with emotions, musical understanding, and wholistic processing. The question arises now: do politically induced trust and distrust need more distinctive abilities to process than brand-related words do? And vice versa, do brand-evoked stimuli need more wholistic and emotional processing? Furthermore, newer research by [51] suggest that congruent stimuli show higher P2a (100–200 ms) and P3 (300–400 ms) amplitudes, implying enhanced processing. At least the secondly mentioned finding of [51] could be replicated here, which allows the assumption that politically induced trust and distrust not only possess higher arousal levels but also seem to be more congruent than brand-related notions. This supports the two-dimensional approach of withdrawal and approach by [52].

Finally, we showed that trust especially differs in its neurophysiological processing among diverse personality factors. Assessed by between-subject ANOVAs, economically induced trust at 780 ms on the right hemisphere seemed to differ between the expression levels of agreeableness and honesty–humility. Even though a categorization of higher and lower trust amplitudes between the three possible values of honesty–humility and agreeableness could not be accomplished due to the inverse problem [53], the findings of this study state that interindividual expressions in these personality facets are relevant for neuronal trust processing. This shows, from a new approach, that dispositional aspects influence in which manner trust is perceived by individuals and why trusting is not universally achieved by the same activities. Further research is needed to obtain a deeper understanding of the neurobiological connection between trust and distrust and of the degree to which these dispositive tendencies influence the interactional trust process.

## 6. Limitations

Even though this paper explored, for the first time, aspects of trust and distrust that have not been stated before, some limitations decrease the validity of this paper. Beginning with the most obvious fact, we assessed the following research question with a very small sample (n = 37), which did not allow for any representative conclusions about the phenomena we explored outside of the used sample range. In other words, the generalizability must be treated with great caution. Furthermore, the sampling followed the snowball principle, which also diminishes the sensitivity of our data [32].

Besides these aspects, we could not solve the inverse problem of the constructs of trust and distrust due to lacking spatial analysis of the phenomena. With this in mind, future research could examine the elaborated constructs of trust and distrust through multiple biological methods (for instance, by mixing fMRI and EEG). This could solve the inverse problem and augment the reliability of the presented findings. Additionally, we used Benjamini–Hochberg correction to diminish alpha inflation, which is an optimal alternative to Bonferroni or Bonferroni–Holm corrections, especially when working exploratively with many hypotheses, but, nevertheless, it shows higher false-positive rates than the conservative alternatives [54].

Moreover, correction concerning fatigue or carry-over effects could only be carried out partially because, especially in quasi-experimental conditions, the control of all confounding variables is not possible. Therefore, a replication of this design, with newly randomized sample-groups, would be recommendable to support and validate the presented findings. Lastly, the validity of ERP studies and the classical RSVP paradigm, as performed in this experiment, seems to be lacking, as shown by newer FRP (fixation-related potentials) and SRP (saccade-related potentials) studies (for instance, [46,55,56]), which allow, through the usage of eye-tracking, higher levels of control concerning active visual attention. This means that there is a probability that some of the accumulated data samples used in the averaging process do not represent active trust/distrust processing.

## 7. Conclusions

The aim of the current study was to support conventional trust theory with neuropsychological evidence. This paper can be seen as an extension of a study presented by the authors at the NeuroIS Congress 2023 on general differences in trust and distrust processing [19], which is currently available as a proceedings volume. This extension specifically lies in the additional analysis of the HEXACO-60 dimensions of honesty–humility and agreeableness and their connection with trust and distrust processing. The results show that while trust and distrust processing differs overall, differences could be observed at electrode F8, 780 ms after stimulus onset, between the chosen personality categories concerning brand names and political institutions. This implies that the intensity of expression in a personality dimension influences the neurophysiological processing of trust.

## Figures and Tables

**Table 1:** Descriptive data of the facet of honesty–humility.

Honesty–Humility Category								
Studied Psychology					Frequency	Percentage	Valid Percentage	Cumulated Percentage
No	Male	A-levels	Valid	Normal distribution	11	91.7	91.7	91.7
				High scores	1	8.3	8.3	100.0
				Total	12	100.0	100.0	
		Higher-education institution	Valid	Normal distribution	3	100.0	100.0	100.0
	Female	Compulsory school	Valid	Normal distribution	1	100.0	100.0	100.0
		A-levels	Valid	Normal distribution	6	66.7	66.7	66.7
				High scores	3	33.3	33.3	100.0
				Total	9	100.0	100.0	
		Higher-education institution	Valid	Low scores	1	33.3	33.3	33.3
				High scores	2	66.7	66.7	100.0
				Total	3	100.0	100.0	
Yes	Male	A-levels	Valid	Normal distribution	3	100.0	100.0	100.0
		Higher-education institution	Valid	Normal distribution	1	100.0	100.0	100.0
	Female	A-levels	Valid	Normal distribution	3	75.0	75.0	75.0
				High scores	1	25.0	25.0	100.0
				Total	4	100.0	100.0	
		Higher-education institution	Valid	Normal distribution	1	100.0	100.0	100.0
**Total**	Valid	Missing	Average Value	Standard deviation	37	0	2.1622	0.4418

**Table 2:** Descriptive data of the facet of agreeableness.

Agreeableness Category								
Studied Psychology					Frequency	Percentage	Valid Percentage	Cumulated Percentage
No	Male	A-levels	Valid	Low scores	1	8.3	8.3	8.3
				Normal distribution	8	66.7	66.7	75
				High scores	3	25.0	25.0	100.0
				Total	12	100.0	100.0	
		Higher-education institution	Valid	Normal distribution	3	100.0	100.0	100.0
	Female	Compulsory school	Valid	Normal distribution	1	100.0	100.0	100.0
		A-levels	Valid	Low scores	1	11.1	11.1	11.1
				Normal distribution	7	77.8	77.8	88.9
				High scores	1	11.1	11.1	100.0
				Total	9	100.0	100.0	
		Higher-education institution	Valid	Low scores	2	66.7	66.7	66.7
				Normal distribution	1	33.3	33.3	100.0
				Total	3	100.0	100.0	
Yes	Male	A-levels	Valid	Low scores	2	66.7	66.7	66.7
				High scores	1	33.3	33.3	100.0
				Total	3	100.0	100.0	
		Higher-education institution	Valid	Normal distribution	1	100.0	100.0	100.0
	Female	A-levels	Valid	Low scores	1	25.0	25.0	25.0
				Normal distribution	1	25.0	25.0	50.0
				High scores	2	50.0	50.0	100.0
				Total	4	100.0	100.0	
		Higher-education institution	Valid	Normal distribution	1	100.0	100.0	100.0
**Total**	Valid	Missing	Average Value	Standard deviation	37	0	2.00	0.6236

**Table 3:** Results of the matched-pairs *t*-tests.

Matched-Pair *t*-Test								
Paired Differences								
	Average Value	Standard Deviation	95% Confidence Interval of the Differences		T	df	Sig. (2-Sides)	Cohen’s d
			Lowest Value	Highest Value				
F7: Brand trust vs. distrust at 330 ms	−0.075	2.389	−0.872	0.721	−0.191	0	0.849	−0.031
F7: Pol. trust vs. distrust at 330 ms	1.786	3.591	0.589	2.983	3.026	36	0.005	0.497
F7: Brand trust vs. distrust at 780 ms	−0.207	2.604	−1.075	0.661	−0.483	36	0.632	−0.079
F8: Pol. trust vs. distrust at 780 ms	0.508	2.552	−0.343	1.359	1.211	36	0.234	0.199
P7: Brand trust vs. distrust at 330 ms	−0.261	2.362	−1.048	0.527	−0.672	36	0.506	−0.11
F7: Pol. trust vs. distrust at 780 ms	0.004	3.297	−1.095	1.104	0.008	36	0.994	0.001
P8: Brand trust vs. distrust at 330 ms	−0.120	2.593	−0.984	0.745	−0.281	36	0.781	−0.04
P8: Pol. trust vs. distrust at 780 ms	−0.052	3.262	−1.140	1.035	−0.097	36	0.923	−0.02

**Table 4:** Results of the Wilcoxon tests.

Wilcoxon Test								
	F7: Pol. Trust vs. Distrust at 780 ms	F8: Brand Trust vs. Distrust at 330 ms	F8: Brand Trust vs. Distrust at 780 ms	F8: Pol. Trust vs. Distrust at 330 ms	P7: Pol. Trust vs. Distrust at 330 ms	P7: Brand Trust vs. Distrust at 780 ms	P8: Pol. Trust vs. Distrust at 330 ms	P8: Brand Trust vs. Distrust at 780 ms
Z	−1.924	−0.581	−2.633	−1.154	−2.105	−1.441	−0.898	−0.747
Asymp. Sig. (2 sides)	0.054	0.561	0.008	0.248	0.035	0.150	0.369	0.455

Note: The red characters symbolize a highly significant result, which was used for the final interpretation.

**Table 5:** Results of the MANOVA procedure.

Multivariate tests						
Effect		Value	F	Treat df	Error df	Sig.
Honesty–Humility Category	Pillai–Bartlett trace ∑	0.864	5322	8000	56,000	0.000
	Wilk’s lambda Λ	0.281	5987	8000	54,000	0.000
	Hotelling’s trace T2	2.046	6649	8000	52,000	0.000
	Roy’s largest root θ	1.752	12,262	4000	28,000	0.000
Agreeableness Category	Pillai–Bartlett trace ∑	0.957	6,422	8000	56,000	0.000
	Wilk’s lambda Λ	0.179	9221	8000	54,000	0.000
	Hotelling’s trace T2	3.839	12,478	8000	52,000	0.000
	Roy’s largest root θ	3.630	25,412	4000	28,000	0.000
Honesty–Humility Category × Agreeableness Category *	Pillai–Bartlett trace ∑	0.920	5968	8000	56,000	0.000
	Wilk’s lambda Λ	0.159	10,191	8000	54,000	0.000
	Hotelling’s trace T2	4.800	15,600	8000	52,000	0.000
	Roy’s largest root θ	4.694	32,857	4000	28,000	0.000

* This operation tries to convey the interaction between Honesty-Humilty and Agreeableness. Note: The red characters symbolize a highly significant result, which was used for the final interpretation.

**Table 6:** Results of the follow-up one-directional ANOVA.

Tests of Between-Subject Effects							
Source		Type III Sum of Squares	df	Mean Square	F	Sig.	Partial Eta-Square
Honesty–Humility Category	F8: Brand trust at 780 ms	94,004	2	47,002	6.130	0.006	0.290
	F8: Brand distrust at 780 ms	75,778	2	37,889	4.806	0.015	0.243
	F7: Pol. trust at 330 ms	45,953	2	22,976	1.858	0.173	0.110
	F7: Pol. distrust at 330 ms	39,873	2	19,936	1.698	0.200	0.102
Agreeableness Category	F8: Brand trust at 780 ms	275,162	2	137,581	17.944	0.000	0.545
	F8: Brand distrust at 780 ms	14,059	2	7,029	0.892	0.421	0.056
	F7: Pol. trust at 330 ms	69,027	2	34,513	2.791	0.077	0.157
	F7: Pol. distrust at 330 ms	54,355	2	27,178	2.315	0.116	0.134
Honesty–Humility Category × Agreeableness Category *	F8: Brand trust at 780 ms	486,926	2	243,463	31.754	0.000	0.679
	F8: Brand distrust at 780 ms	5678	2	2839	0.360	0.701	0.023
	F7: Pol. trust at 330 ms	37,001	2	18,501	1.496	0.240	0.091
	F7: Pol. distrust at 330 ms	20,000	2	10,000	0.852	0.437	0.054
Error	F8: Brand trust at 780 ms	230,016	30	7667			
	F8: Brand distrust at 780 ms	236,487	30	7883			
	F7: Pol. trust at 330 ms	370,943	30	12,365			
	F7: Pol. distrust at 330 ms	352,219	30	11,741			

* This operation tries to convey the interaction between Honesty-Humilty and Agreeableness. Note: The red characters symbolize a highly significant result, which was used for the final interpretation.

## Data Availability

Data are contained within the article.

## Appendix A

**Table A1:** Benjamini–Hochberg Correction.

Benjamini–Hochberg Correction		
*p*-Values	Ranks (i)	P_i_ ≤ (i/31) ×0.05
0.001	1	0.00161290
0.001	2	0.00322581
0.001	3	0.00483871
0.001	4	0.00645161
0.001	5	0.00806452
0.005	6	0.00967742
0.006	7	0.01129032
0.008	8	0.01290323
0.015	9	0.01451613
0.035	10	0.01612903
0.054	11	0.01774194
0.077	12	0.01935484
0.15	13	0.02096774
0.116	14	0.02258065
0.173	15	0.02419355
0.234	16	0.02580645
0.2	17	0.02741935
0.24	18	0.02903226
0.248	19	0.03064516
0.369	20	0.03225806
0.421	21	0.03387097
0.437	22	0.03548387
0.455	23	0.03709677
0.506	24	0.03870968
0.561	25	0.04032258
0.632	26	0.04193548
0.701	27	0.04354839
0.781	28	0.04516129
0.849	29	0.04677419
0.923	30	0.04838710
0.994	31	0.05000000

Note: The red-filled bar symbolizes the termination of possible significant corrections.

**Table A2:** Box’M test for covariance matrix homogeneity.

Box’M Test for Covariance Matrix Homogeneity	
Box’ M	30.490
F	0.951
df1	20
df2	510.561
Sig.	0.522

**Table A3:** Levene test for variance homogeneity.

Levene Test for Variance Homogeneity					
		Levene Statistic	df1	df2	Sig.
eco_V_F8_779	Based on mean	0.895	4	30	0.479
	Based on median	0.897	4	30	0.478
	Based on median and with adjusted df	0.897	4	22,941	0.482
	Based on trimmed mean	0.878	4	30	0.489
eco_M_F8_779	Based on mean	2.424	4	30	0.070
	Based on median	1.820	4	30	0.151
	Based on median and with adjusted df	1.820	4	19,189	0.166
	Based on trimmed mean	2.404	4	30	0.072
pol_V_F7_331	Based on mean	4.150	4	30	0.009
	Based on median	3.039	4	30	0.032
	Based on median and with adjusted df	3.039	4	23,550	0.037
	Based on trimmed mean	3.940	4	30	0.011
pol_M_F7_331	Based on mean	1.541	4	30	0.216
	Based on median	1.425	4	30	0.250
	Based on median and with adjusted df	1.425	4	18,672	0.265
	Based on trimmed mean	1.523	4	30	0.221

**Table A4:** Normal distribution of agreeableness.

Testing Normal Distribution							
Agreeableness Category		Kolmogorov–Smirnov			Shapiro–Wilk		
		Statistic	df	Significance	Statistic	df	Significance
F8: Eco. trust at 780 ms	1.00	0.151	7	0.200 *	0.932	7	0.571
	2.00	0.174	23	0.068	0.923	23	0.077
	3.00	0.387	7	0.002	0.627	7	0.001
F8: Eco. distrust at 780 ms	1.00	0.193	7	0.200 *	0.940	7	0.636
	2.00	0.079	23	0.200 *	0.968	23	0.644
	3.00	0.230	7	0.200 *	0.894	7	0.298
F7: Pol. trust at 330 ms	1.00	0.199	7	0.200 *	0.942	7	0.661
	2.00	0.225	23	0.004	0.869	23	0.006
	3.00	0.210	7	0.200 *	0.906	7	0.367
F7: Pol. distrust at 330 ms	1.00	0.240	7	0.200 *	0.814	7	0.056
	2.00	0.115	23	0.200 *	0.962	23	0.506
	3.00	0.165	7	0.200 *	0.924	7	0.497

* *p* < 0.001.

**Table A5:** Normal distribution of honesty–humility.

Testing Normal Distribution							
Honesty–Humility Category		Kolmogorov–Smirnov			Shapiro–Wilk		
		Statistic	df	Significance	Statistic	df	Significance
F8: Eco. trust at 780 ms	2.00	0.109	29	0.200 *	0.985	29	0.938
	3.00	0.273	7	0.125	0.809	7	0.050
F8: Eco. distrust at 780 ms	2.00	0.110	29	0.200 *	0.973	29	0.641
	3.00	0.271	7	0.129	0.837	7	0.094
F7: Pol. trust at 330 ms	2.00	0.135	29	0.188	0.955	29	0.239
	3.00	0.137	7	0.200 *	0.943	7	0.664
F7: Pol. distrust at 330 ms	2.00	0.119	29	0.200 *	0.973	29	0.650
	3.00	0.120	7	0.200 *	0.964	7	0.856

* *p* < 0.001.

**Table A6:** Normal distribution of the selected electrodes for matched-pair t-tests analysis.

Testing Normal Distribution						
Neuronal Potentials	Kolmogorov–Smirnov			Shapiro–Wilk		
	Statistic	df	Significance	Statistic	df	Significance
F7: Eco_trust/distrust_330	0.081	37	0.200 *	0.971	37	0.424
F7: Pol_trust/distrust_330	0.119	37	0.200 *	0.957	37	0.166
F7: Eco_trust/distrust_780	0.122	37	0.183	0.971	37	0.426
F7: Pol_trust/distrust_780	0.189	37	0.002	0.837	37	0.000
F8: Eco_trust/distrust_330	0.187	37	0.002	0.886	37	0.001
F8: Pol_trust/distrust_330	0.158	37	0.020	0.842	37	0.000
F8: Eco_trust/distrust_780	0.211	37	0.000	0.655	37	0.000
F8: Pol_trust/distrust_780	0.130	37	0.114	0.953	37	0.123
P7: Eco_trust/distrust_330	0.083	37	0.200 *	0.973	37	0.491
P7: Pol_trust/distrust_330	0.207	37	0.000	0.819	37	0.000
P7: Eco_trust/distrust_780	0.250	37	0.000	0.589	37	0.000
P7: Pol_trust/distrust_780	0.151	37	0.033	0.960	37	0.203
P8: Eco_trust/distrust_330	0.087	37	0.200 *	0.983	37	0.829
P8: Pol_trust/distrust_330	0.260	37	0.000	0.787	37	0.000
P8: Eco_trust/distrust_780	0.269	37	0.000	0.560	37	0.000
P8: Pol_trust/distrust_780	0.140	37	0.064	0.970	37	0.400

* *p* < 0.001.

**Table A7:** All word stimuli that were used for the experiment.

Adidas	Puma	Nike	Apple	Samsung
Huawei	ÖMV	Voestalpine	Strabag	WienEnergie
Verbund	RalphLauren	CalvinKlein	Dior	Zara
C&A	H&M	Gucci	Versace	LouisVuitton
Burberry	Rolex	Omega	MarcoPolo	Esprit
GAP	MichaelKors	Cartier	Swarowski	Peek&Cloppenburg
BILLA	DM	Bipa	Spar	Hofer
Penny	Lidl	Tesla	Bayer	Johnson&Johnson
Pfizer	Moderna	Genericon	AstraZeneca	Nestle
CocaCola	Pepsi	Starbucks	Chanel	Google
Yahoo	LinkedIn	Nescafe	Ford	Toyota
Disney	Netflix	Amazon	Microsoft	IBM
SpaceX	Prada	Intel	Visa	L’oreal
Firefox	Pringles	Ben&Jerrys	Knorr	TommyHilfiger
Sony	LG	Ikea	Marlboro	Audi
PayPal	Ottakringer	Heineken	Lego	Xiaomi
ÖVP	SPÖ	Neos	FPÖ	Grüne
BierPartei	KPÖ	MFG	Arbeiterkammer	ÖGK
Nationalrat	Bundesrat	Parlament	Innenministerium	Außenministerium
Gesundheitsministerium	Verteidigungsministerium	Finanzministerium	Justizministerium	Umweltministerium
Kulturministerium	Landwirtschaftsministerium	Wirtschaftskammmer	Landwirtschaftskammer	Ärztekammer
Apothekerkammer	Zahnärztekammer	Rechtsanwaltskammer	Industriellenvereinigung	Verfassungsgerichtshof
Verwaltungsgerichtshof	ObersterGerichtshof	WKStA	U-Ausschuss	DerStandard
DiePresse	HeuteZeitung	ÖsterreichZeitung	Kurier	OE24
ORF	KroneZeitung	KleineZeitung	SalzburgerNachrichten	WienerZeitung
FalterZeitung	NewsZeitung	AugustinZeitung	DieTagespresse	Antifa
DieIdentitären	IslamischerStaat	DieGrauenWölfe	AlQaida	Hamas
Greenpeace	PETA	WWF	Global2000	Alpenverein
VierPfoten	Bundeskanzleramt	Bundespräsidentenamt	JUNOS	AktionsGemeinschaft
VSSTÖ	GrasPartei	RingFreiheitlicheStudierende	KSV	Cartellverband
Pensionistenverband	Bauernbund	Arbeitnehmerbund	Wirtschaftsbund	Seniorenbund
KarlRennerInstitut	PolitischeAkademieÖVP	FreiheitlichesBIldungsInstitut	GrüneBildungswerkstatt	NEOSLab
